# More Than the Sum of Multiple Care: Ambivalence in Sandwich Care

**DOI:** 10.1111/1468-4446.13202

**Published:** 2025-03-21

**Authors:** Junko Yamashita, Naoko Soma

**Affiliations:** ^1^ School of Sociology, Politics and International Studies University of Bristol Bristol UK; ^2^ International Graduate School of Social Science Yokohama National University Yokohama Japan

**Keywords:** gender and policy, motherhood, older adult care, sandwich care, sociological ambivalence

## Abstract

A growing population in economically developed societies are simultaneously providing childcare and older adult care, or sandwich care. The existing studies reveal that sandwich carers are more physically, mentally and financially challenged than those providing dyadic care. This article explores an understudied area of sandwich care and ambivalence. Ambivalence encompasses the difficulties, challenges, and range of feelings, including guilt, anger, isolation, sense of duty, fulfilment and many others that sandwich carers' experience. Building on the existing sociological approach to ambivalence, this paper proposes a theoretical framework for delineating the entangled structural and relational webs where sandwich carers' experiences and negotiations are situated. Our theoretical framework captures the *temporal*, *socially structured* and *policy‐contextual* properties of ambivalence. We argue that ambivalence arises from historical and prospective family relationships (temporal) that intersect with the gendered expectations for parenting and family responsibility of adult social care (socially structured), which further intersects with care policy and available care services (policy contextual). The three qualities of ambivalence influence each other in multiple ways. Socially structured and temporal qualities of ambivalence can influence sandwich carers' access to and experience of using care services, but the social arrangement of care can also increase or mitigate ambivalence in sandwich care arising from them. While we illustrate this by drawing on considerable evidence from Japan, we argue that our study provides a useful theoretical framework attuned to understanding the experience of such carers in diverse social and cultural contexts.

## 1 Introduction

A growing proportion of women in economically developed societies are starting a family much later in life. In 2021, the average age to give birth for the first time was over 30 years old in most OECD countries, an increase of 4.5 years from 1995 (OECD 2023a). This trend has created a crossover period in women's life course when they simultaneously provide childcare and older adult care, or sandwich care (Burke and Calvino 2017). This is an emerging yet under‐researched social and demographic phenomenon. Taking the UK and Japan as examples, the Office for National Statistics (ONS) estimated 3% of UK adults are sandwich carers, with 72% aged between 35 and 54 years old. This section of the population is reported as having poorer mental and physical health as well as greater financial struggles than those who provide either childcare or older adult care alone (Office of National Statistics 2019). In Japan, a survey which included adults with a co‐living child of university age or younger, estimated 22.9% of adults were sandwich carers (Sony Life Insurance 2024) with an average age of 44.3 years. Again, Japanese data reveals that sandwich carers feel mentally, physically and financially burdened (*ibid*). In both societies, women make up the overwhelming majority of sandwich carers: 62.4% in the UK and 67% in Japan.

This paper explores what characterises difficult experiences of sandwich carers to make them feel more burdened than those who provide dyadic care. These difficulties are captured in data collected from interviews with sandwich carers, who expressed ambivalent feelings about providing everyday sandwich care. Carers brought up their ambivalent feelings when describing the ways in which they prioritised between taking care of children and older parents, and when their desire to provide more or less care to either children or older parents, or both, were not realised in the everyday practice of providing sandwich care. Ambivalence is used in this context to include the practical challenges and range of sometimes conflicting feelings these carers experience. By extending the existing sociological approach to ambivalence, this paper presents a new theoretical framework to detail the entangled structural and relational factors influencing the experiences and negotiations of sandwich carers. Our analysis reveals that their ambivalence has three properties: *socially structured*, *temporal* and *policy‐contextual*. Ambivalence was formed by social expectations associated with motherhood and family responsibilities for older adult care, intersecting with historical and prospective family caring relationships and further intersecting by the social arrangement of care—care policy frameworks and available social care services. While we draw extensively on evidence from Japan and consider its specific contexts to understand the experiences of sandwich carers, we argue that our discussion transcends uniquely Japanese situations and provides a valuable theoretical framework for understanding the experiences of carers involved in multiple care.

We first provide a brief review of the relevant scholarship on sandwich care and sociological ambivalence, and present our theoretical framework of ambivalence. We then discuss family roles in and the policy context of child and older adult care. After introducing research methods, we apply our theoretical approach of ambivalence to the analysis of sandwich carers' experiences. In conclusion, we discuss our findings: entangled ambivalence when women provide multiple care, that has, socially structured, temporal and policy contextual properties.

## 2 Sandwich Care and Sociological Ambivalence

### 2.1 Sandwich Care

We adopt the definition of care proposed by Daly (Daly 2021, 113) as a welfare‐related activity focused on practices oriented to meeting perceived needs, which is situated at the intersection of need, relations/actors, recourses and values. Care thus includes the management of care, such as gathering appropriate information, communicating with care professionals and organising resources to provide care as appropriately as possible, as well as travelling for care. The existing studies argue that sandwich care is more challenging than dyadic care, because of the difficulties of simultaneously managing childcare and older adult care (Mitchell 2014), which adversely impacts carer well‐being and resources (Rubin and White‐Means 2009; Tan 2018). The difficulties and contradictions include: having to respond to different types of care needs (Halinski et al. 2018), experiencing clashing roles as mother and daughter (Železná 2018), and managing multiple tasks often within time constraints of daily life doing paid work (Steiner and Fletcher 2017).

### 2.2 Sociological Ambivalence

A sociological approach to ambivalence provides a useful analytical framework to capture these difficulties in sandwich care, which have previously been mostly studied empirically. Ambivalence has been developed as a sociological concept to examine the experiences of mixed feelings (including contradictions, tensions and/or dissonance) by identifying them arising in individual transactions with others, located in dynamic relationships within social structures. Applying the sociological ambivalence approach, this paper provides a theoretical framework that identifies the main qualities of ambivalence, giving a sociological tool to comprehend sandwich carers' experiences.

The first to discuss sociological ambivalence were Merton and Barber (1976). They conceptualised ambivalence sociologically, as opposed to psychologically, by examining it as the product of conflicting norms and counter‐norms associated with particular social positions (Merton and Barber 1976). While social norms and role expectations were the subjects of their analysis, other early studies of sociological ambivalence predominantly applied the concept to the analysis of the psychological state of the individual, investigating how ambivalence as ‘mixed feelings’ is experienced and what is done to resolve it (Smelser 1998). Subsequent work pays more attention to social structures that construct ambivalence at the individual level (Luscher and Pillemer 1998) and the links between individual agency and social structure (Connidis and McMullin 2002; Lorenz‐Meyer 2001; Hillcoat‐Nallétamby and Phillips 2011; Pillmer et al. 2019; Järvinen and Luckow 2020). This study will focus on the theoretical approach of Connidis and McMullin (2002) and Hillcoat‐Nallétamby and Phillips (2011). They proposed a theoretical framework that applies the concept of ambivalence beyond the dichotomy of individual and structural level‐analysis, to individual interactions (Connidis and McMullin 2002) and transactions (Hillcoat‐Nallétamby and Phillips 2011) with others. Thus, they made key contributions to the conceptual development of sociological ambivalence. We extend their theoretical framework further to situate sandwich carers' ambivalence in the web of their transactions and relationships with others, social expectations and the social arrangement of care.

Connidis and McMullin (2002) propose an ambivalence concept as a vehicle for connecting individuals, their relationships, social institutions and social structure. Social structure, being defined as structured social relations such as gender, class and ethnicity, is composed of interlocking sets of social relations that privilege or disadvantage certain groups. It encourages and enforces particular commitments by specific groups and distributes resources differently in the arrangement of social institutions (Connidis and McMullin 2002; Connidis 2015); a typical example is the gendered distribution of care in the family. Combining such critical sociology's view of social structure with symbolic interactionism, they argue that social structural contradictions are manifested only through ‘social actors who make decisions about and negotiate courses of action’ in their interaction with others (*ibid*, 563). Connidis and McMullin (2002, 559) thus conceptualise ambivalence as ‘structurally created contradictions that are experienced by individuals in their interaction with others’.

Hillcoat‐Nallétamby and Phillips (2011) argue that the relational approach is critical for the analysis of ambivalence. They emphasise that ambivalence arises in transactional processes, where agency, power and social structure gain meaning and substances. Dépelteau (2015) distinguishes between interactions and transactions, arguing that transactions are not simply interactions as ‘the action of A cannot be disconnected from the action of B, and vice versa’ (*ibid*, 56). An example of when ambivalence might arise is with the shift in power from ageing parents to adult children as the former become less ‘independent’ and need care (Hillcoat‐Nallétamby and Phillips 2011). They elaborate on the essential element of a relational approach to ambivalence:

Ambivalence is situated at the nexus of complex, dynamic figurations of relational experiences which are patterned by the temporality of relational histories and the socially structured dimensions of human existence (Hillcoat‐Nallétamby and Phillips 2011, 206)

With the relational sociological approach, they widen the analytical scope of ambivalence to temporal properties, which views relational transactions across historical periods and individual life courses. Ambivalence includes historical and prospective dimensions: the former being characterised by relational transactions over a long period, and the latter capturing individuals' concerns with ‘trying to anticipate’ the future generated by potential situations, needs and relationships (Hillcoat‐Nallétamby and Phillips 2011).

### 2.3 Our Theoretical Framework of Ambivalence

Our theoretical framework to ambivalence brings together both Connidis and McMullin (2002) and Hillcoat‐Nallétamby and Phillips (2011) and extends to include the social arrangement of care. We locate ambivalence within individuals' experiences of their transactions with others, which are shaped by and shape social structures. In addition, however, we will emphasise the importance of the social arrangement of care, such as relevant policy frameworks and available social care services, in the dynamic formation of this ambivalence. The studies adopting a sociological ambivalence approach have not explicitly included social policy in their analysis. Notable exceptions are Neuberger and Haberkern (2014), Järvinen and Luckow (2020) and Yamashita and Soma (2024) in which ambivalence arising from tensions between policy frameworks and social expectations are discussed. However, these studies do not provide a theoretical framework to delineate the ambivalent experience of carers.

**FIGURE 1:**
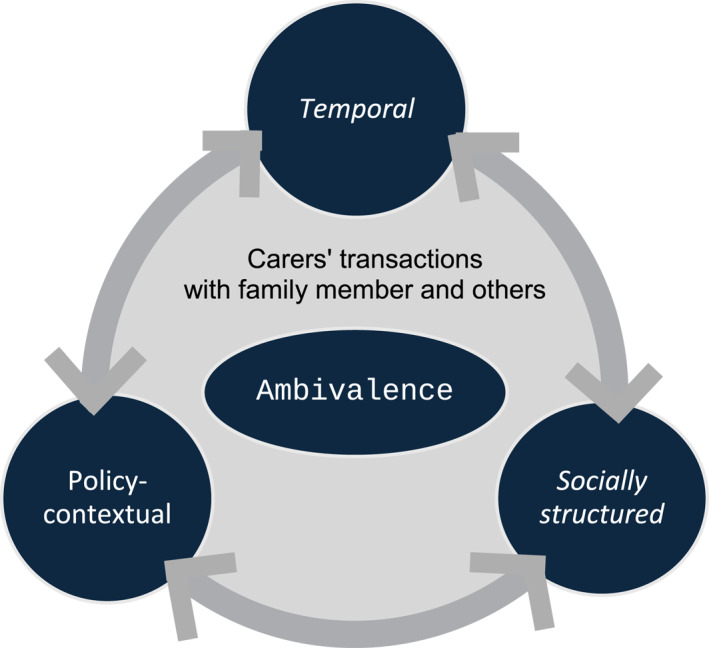
Our theoretical framework of ambivalence.

Our theoretical framework, then, captures the *socially structured*, *temporal* and *policy‐contextual* properties of ambivalence (See Figure 1). We conceptualise ambivalence as affective manifestations of structural contradictions experienced by individuals in transactions with others. We examine sandwich carers' ambivalence with gendered expectations for parenting and adult social care (*socially structured*). Their ambivalence has the temporal property that ambivalence in the present is better understood in patterned relational transactions formed over a long period of time and by the anticipated future of the relations (*temporal*). Policy contexts increase or mitigate their ambivalence (*policy contextual*), but socially structured and temporal qualities of ambivalence can also influence ambivalence arising from policy contexts. The three qualities of ambivalence influence each other in multiple ways. Our three‐fold theoretical approach to ambivalence places carers' affective experiences of ambivalence in carers' transactions with family members and others within temporal family relationships extending from the past into the future, and the present contexts of social expectations and of the social arrangement of care. The following analysis will illustrate how our theoretical framework illuminates sandwich carers' experiences. A brief discussion on policy development and social expectations of care in Japan is provided to contextualise the analysis in the next section.

## 3 Policy and the Gendered Context of Family Care: The Expansion of Publicly Funded Care and the Gendered Division of Care

Strong family roles and the sustained gendered division of caregiving in Japan and East Asia have been extensively discussed (Liu and Yamashita 2020). The Japanese government places expectations on families to finance and provide care, and on women to provide unpaid family care through policy measures such as spousal tax deductions (Soma and Yamashita 2020). However, since the 1990s, the demographic trends of ageing populations and low fertility rates have promoted a policy shift for the government to take more financial responsibility for and expand statutory roles in the organisation of care for both older people and children (Yamashita and Soma 2023). Publicly‐funded social care services have significantly expanded since the 1990s; Japanese public spending on childcare services (OECD 2023a) and long‐term care (OECD 2023b) are now well above the OECD average.

Although Japan has seen a significant expansion of childcare services since the 1990s (Estévez‐Abe and Naldini 2016), the recent expansion of public‐funded childcare through marketisation has not considered gender equality or supporting families with children as an end in itself. Instead, the childcare policy development was justified as a means to revitalise the economy and increase the fertility rate to tackle the effects of a shrinking and ageing population (Yamaura 2020), expecting women to fulfil the responsibility of both mothers and workers.

Gendered expectations and practices regarding older adult care differ from those in childcare. A significant development in older adult care was the implementation of the Long‐Term Care Insurance (LTCI) Act in 2000. Its policy aim was set to shift care responsibilities from the family to the government while also controlling healthcare costs for older people (Yamashita 2011), and it has removed some of the responsibility for adult social care from the family. The Japanese LTCI policy is generous in terms of its coverage and types of services provided. It provides both institutional and home care services, and both physical (e.g., dressing, helping with eating) and instrumental (e.g., shopping and cleaning) care to those judged to be in need of support. The implementation of LTCI has influenced who provides care to older parents. The ‘traditional’ norm that a daughter‐in‐law is obliged to take care of her parents‐in‐law has been significantly weakened. The apparent trends are: (1) the decrease in co‐living carers and the increase of non‐co‐living children and professional care workers (15.7%), and (2) the decrease of daughters‐in‐law and the increase of male spouses as main carers (Ministry of Health Labour and Welfare 2023). In practice, however, the LTCI scheme alone does not meet the care needs of all older people (Mori 2023). Thus, family care and care services outside the scheme are required to support the ‘autonomous’ living of older people. Moreover, subsequent reforms have narrowed the range of services, and tightened eligibility, resulting in a shift of the responsibility for care from the state back to the family (Fujisaki 2009; Shimoebisu 2015).

In summary, despite the substantial expansion of publicly funded care services, Japanese social policy has continued to uphold ‘the family household as the locus of care, and reinforced the role of wives, mothers, and in eldercare, daughters, as those who provide for young and elderly family members’ (Shire and Nemoto 2020, 443). Thus, it reinforces gendered expectations and division of paid work and unpaid care work. Miura (2015) points out that the gendered role division embodied in the role of mother is the basis of the gendered dual structure of the labour market in Japan which guarantees employment and labour force flexibility. With these policy frameworks and the labour market structure, the gendered division of care and domestic work is sustained: gender gaps in both paid and unpaid work are significant in Japan (Kan et al. 2022), and Japanese men contribute the least to childcare and domestic work among the OECD countries (OECD 2018).

## 4 Finding Sandwich Carers: Research Methods

To unpack the web of sandwich carers' experiences with the sociological ambivalence approach, we draw on the analysis of qualitative data from our research project, ‘Comparative Analysis of Care Regimes and Dual Responsibility of Childcare and Elderly Care in East Asia’ (see Yamashita and Soma 2023, for more detailed research methods of the project). This project was designed to understand the everyday practice and experience of sandwich carers, and how these are shaped by and shape related social policy in Hong Kong, Japan, South Korea and Taiwan. This paper focuses on the data from Japan (Yamashita and Soma 2023). Semi‐structured interviews (60) and focus group interviews (13) were conducted with women in their 30s, 40s and 50s with co‐living children in education across Japan between 2012 and 2023. We introduced an expanded conceptualisation of care to include activities focused on meeting perceived physical, emotional and financial needs of dependent adults and children, as well as travel and managing care remotely. Based on this definition, respondents who identified themselves as providing both care for older people and childcare were invited to an interview, regardless of older people's level of care needs judged by the Japanese LTCI policy. Semi‐structured interviews were conducted in person or by phone with one of the authors. Interview questions were centred around how and why they became sandwich carers, their everyday experience of providing such care, and what support they received. Interviews, coding and initial thematic analysis of qualitative data were shared equally by both authors. Based on the initial thematic analysis, key themes were identified, including priorities, support networks, care service usage, financial and emotional burdens, and family relationships. Ambivalence emerged as an overarching theme. Many participants also articulated ambivalence especially related to an event when they had to prioritise care for children or their parents.

The following discussion is based on the analysis of all the qualitative data, but in the limited scope of this paper, it will focus on the data from four interviews to incorporate the interviewees' respective care experiences and family relationships before engaging with sandwich care. These four interviews were chosen to include key variations in sandwich care that the analysis identified as significantly influential (Soma and Yamashita 2020): different levels of older adult care needs, number and age of children, living arrangements and employment status (Table 1).

**TABLE 1:** The basic information of selected interviewees.

Pseudonym	Mai	Emi	Tomoko	Fumi
Age	35	37	39	35
Who they provide older adult care	Mother	Father	Father	Mother and Father
Level of adult care needs	Level 4	Level 2	Level 1	Level 2 (mother) and level 5 (father)
No. of children and age	9, 4 years and 10 months old	6 and 2 years old	9, 6 and 4 years old	2 years old
Living arrangement (distance from parents' house)	Living together	10‐min walk	2‐h drive	3‐h flight
Use of childcare services	Private kindergarten (4 years old)	None	After school club (9 and 6 years old), day nursery (4 years old)	Day nursery
Use of adult social care services	Day care service (2/a week), home helper (3/a week), nurse visit (1/a week), bath on wheels (1/a week), rental bed	Day care service (2/a week)	Day care services (2/a week), home helper (3/a week) nurse visit (1/a week), dentist visit (1/a week)	Home helper (3/ a week)
Employment status	Stay‐at‐home mother	Stay‐at‐home mother	3 part‐time jobs	Full‐time employment

## 5 Analysing Ambivalences in Sandwich Care

### 5.1 Mai

Mai is an only child and lives with and cares for her mother, whose needs are judged as severe under the LTCI scheme: she has diabetes, is almost sightless and uses a wheelchair. Mai has three children: a primary school‐aged child, a toddler and a baby. Mai was a kindergarten teacher, but left her job when her father required end‐of‐life care after a cancer diagnosis, and she was about to get married. Mai recalls that she had been interested in care‐related jobs since childhood. She had strong expectations of herself as a mother who takes good care of children. As an only child, Mai says that she had no other option but to care for her own mother, but also she feels that she owes her parents.

I had a very serious illness when I was a child, and I had to have surgery and spend a long time in hospital. I think I gave my parents a hard time, and that's why I have to look after her [my mother] myself now. I've seen my father's back, so I think that's the way I should be.

In addition, she had seen how ‘devoted’ her father was in caring for her mother, so she wanted to respect his wish that she looks after her mother. This sense of family duty of care made her reluctant to use the care service for which her mother was eligible. Despite the severity of her needs, her mother only used home nursing and bathing services once a week until just before the interview. Mai said it was ‘hard to get through each day’. She considered using childcare support only in ‘emergencies’ when she cannot take care of both her mother and her children, but childcare services are not immediately available, so they were not useful for her. It is also financially difficult to use regular childcare services because ‘it is not for work I need childcare’. She sought a nursing home for her mother, but there were none suitable within commuting distance (her mother needs dialysis), and Mai has given up on the possibility. ‘So I have ended up carrying them [my children] in the front and back by myself and watching over my mother’. She was doing all of the mother's care and childcare almost entirely on her own. Her husband does little with daily childcare and domestic chores, as he is ‘busy with work’ and ‘away from home’. Mai says, ‘if he is around, I have a luck, but I live on the assumption that he is not around’.

On the recommendation of the care manager,^1^ who saw Mai's mental and physical exhaustion, she decided to use care services under the LTCI scheme. Her mother has started going to daycare three times a week, allowing Mai to spend more time with her children. However, she feels sorry for her mother.

The helper comes in the morning and does things like brushing my mother’s teeth and things like that. When it comes to dialysis… a care taxi comes and takes my mother. When these things happen, I started to feel that all I do is keep an eye on her, and I'm not really caring for her.

However, she is sure that raising her children is a higher priority. So, there is a tremendous ambivalence—‘I can’t decide which comes first’. Although Mai considers her children to be the most important, in her daily life, she thinks that they often come second and she ‘often feels sorry for them’.

Using the available care services or the lack of desired care services increases Mai's ambivalence, which are entangled with gendered social expectations and family responsibility of care, and with the past family relationships. Mai had high expectations of herself as a mother, expressed through her acceptance that her husband does little childcare. Her sense of family responsibility for taking care of her mother is rooted in the caring relationships she experienced within her family. While she tries to meet both role expectations as a daughter and a mother and fulfil her responsibilities, in practice, she experiences physical and emotional difficulties. Admitting the difficulties of providing sandwich care, Mai accepted the care manager's recommendations to use eligible care services. However, using long‐term care services leads to her ambivalence that she is not caring for her mother as her father wished and not properly fulfilling her role as a daughter. She also feels ambivalent about the fact that, contrary to her belief that her children have priority, they are often second for receiving her care in everyday life.

### 5.2 Emi

Emi is in her late 30s and cares for her father, who is in his early 70s, with a level of care needs judged to be mild. He is half‐paralysed and has mild dementia due to a stroke. She visits her father at her parents' home, a 10‐min walk away, almost every day and helps him go to a day care centre twice a week. Emi has two children, aged six and two. She left her job when her first son was born to focus on caring for him until he reached primary school age. Emi took on the role of main carer for her father, as her mother, who is in her late 60s, wanted to continue to work and was away during the day. Emi has an older sister who has a full‐time job and lives 1 hour's drive from their parent's house. As Emi lives closer to her parents and does not have a full‐time job, she felt that she had no choice but to take care of her father. Leaving her job to focus on raising her children has partly led Emi to become the main carer for her father rather than her sister, who has a full‐time job as a ‘legitimate excuse’ (Finch 1989).

Emi's father began needing care soon after her second son was born. She feels that caring for her father and her son is difficult and limited. When asked if she had thought about leaving her son in childcare, Emi said that there was no suitable place for him, referring to the fact that a day nursery providing temporary childcare is on the other side of town and that he needs to take a considerable number of items, such as bedding. However, besides these practical difficulties, she also feels hesitant about leaving her child at a day nursery.

I stayed at home. … I left my job because I wanted to be with my children all the time, so it really, how should I put it, really didn’t work out the way I wanted it to. If I had to leave my children with someone else, then it would have been better not to leave my job in the first place, put my child in a day nursery, and to have a home helper to look after my parents.

Her father does not use all the services he is entitled to under the LTCI scheme. Rather than being financially limited, he wants to be taken care of by his daughter (according to Emi). Her relatives also pressure her to ‘care’ for her father more as she lives close to her parents. They think taking care of her father is ‘the family's responsibility’. She also wanted to take care of him by herself. Emi respects her father, who used to manage most household chores and supported the whole family. Emi feels that she is ‘caught between childcare and elderly care’ and ‘cannot do either of them satisfactorily’. Her ambivalence is most expressed as worries about her children's present and future.

I feel very sorry for him [her second son] because we always move around with time pressure. I am worried about how that will affect my son’s personality development in the future.

At the same time, Emi feels that she would be better able to care for her father if she did not have a second child, or if the children were older. However, she also feels guilty about feeling this way towards her children. Emi's husband supports her by giving her father a lift to his acupuncture appointment every Saturday, and spending time (mainly playing) with children at the weekend. Emi thinks asking any more from him is too much, even to listen to her daily struggles.

The temporal quality of ambivalence intersected with gendered expectations and family responsibility of care form Emi's ambivalence. Emi wants to give back to her father, who took care of his family in the past, but caring for her children limits her ability to care for him as much as she wants. Her involvement with her father's care makes her feel that she is not performing what mothers should do—focussing on caring for her children. She worries that this may have a negative influence on her son's future. Gendered expectations and family responsibility of care also intersect with the social arrangement of care, forming Emi's ambivalence. While care services are available, the use of them conflicts with the mother's role that Emi wants to practice and the family‐centred care that Emi, her father and her relatives value.

### 5.3 Tomoko

Tomoko is in her 30s and cares for her father in his early 70s. He lives alone in a village, a 2‐h drive from Tomoko's house in a city. He had a brain stroke, which left him with aphasia and mild physical disability. His doctor recommended that he lives in a nursing home, but he escaped from the hospital to his home and insisted on staying there. Tomoko supports his strong wish to stay at home, ‘against all other family members’ opinion’. Her father lives independently, receiving multiple services under the LTCI scheme (see Table 1). Tomoko arranged all the services so that someone could visit him at least once a day. She is raising three children, aged 9, 6 and 4. Tomoko has three part‐time jobs. Seeing how her father took care of her grandmother made her think that she should take care of him in turn. Tomoko feels caught between being told by both those who relate to her father (neighbours and relatives) and those who relate to children (teachers and mother's friends) that she should ‘look after’ her father or children more.

Each of them says “take care of them a bit more”, “a bit more” to me, but they have never been in my shoes. I want to take care of them more, but I cannot do it! For my children, I want to spend more time with them, I want to help them with things, like help them with their homework, I feel frustrated that I cannot do that…. Other children do various extra‐curricula, but those children who don’t do extra‐curricula, cannot do those things, like I feel an education gap, I strongly feel it …. Even though children say they want to join a football club, if their parents don’t have a space in their financial situation or time, even though children say they really want to do it, parents cannot let them do it. …

Tomoko recognises that she cannot care for both her children and father at the same time and attempts to resist the social expectations that she should provide more care as a daughter and mother (‘I want to take care of them more, but I cannot do it!’). Tomoko juggles three part‐time jobs not only to support her family but also to save money to finance her father's future care needs. He is using up his savings to pay for the care services^2^ that he currently receives. She also plans to visit him more often. For that, ‘the cost of petrol will not be insignificant’. The financial pressure of her unpredictable father's future care cost meant she has had to prioritise her work and thus sacrificed both her time with her father and children as well as the money for their extra‐curricular lessons. Tomoko expresses her ambivalent feeling, saying ‘I just want to have more time. I want time with my children, and with my father’.

Tomoko's husband does not share any housework or caring for his children or her father. He also does not share childcare costs with her.

He hadn't yet spent any time alone with our youngest, because my husband never took care of children. … I asked him to look after them when my father had the stroke, but he said he couldn't.

Her parents‐in‐law help her with nursery and afterschool pickups while she is juggling part‐time jobs, as they know that their son does not do anything at home. However, Tomoko thinks they do not give good discipline to children. When her children stayed with them for a couple of weeks while she was with her father in hospital, ‘they left children as they were, so they got bad tooth decay!’.

The temporal quality of ambivalence has strong presence in Tomoko's case, though the financing of care is a central issue. Publicly funded older adult care and childcare enable her to manage sandwich care and paid work, but Tomoko's financial preparation for her father's future care need conflicts with her desire to spend time and provide educational opportunities for her children, which she thinks may potentially put her children’s future at a disadvantage. Tomoko is also reluctant to leave her children with her parents‐in‐law. Her husband's lack of financial or practical contribution to childcare increases Tomoko's ambivalence as she believes juggling three jobs is the main source of her ambivalent feeling. Instead of negotiating with her husband over childcare, she spends all her spare time studying for her qualifications—IT skills and nursing—so that she can have a better‐paid job to create more time with her children and father.

### 5.4 Fumi

Fumi, an only child in her 30s, is the main carer for her parents, who are also supported by professional care workers. She lives in an Southern Island because of her husband's work, which is a 3‐h flight from her parents' house near Tokyo. She has a two‐year‐old daughter. At the time of the interview, Fumi had taken 3 months' leave from her full‐time job, and was staying at her parents' house with her daughter. She was searching for a nursing home for her father, who had dementia and was in hospital after surgery for cancer. She was also there to help her mother convalesce after a stay in hospital and to discuss her mother's care plan with her and her care manager. Fumi usually supports her parents' daily life remotely, communicating with them, their care manager and visiting nurses. However, if something happens, she feels that she has to return to her parent's home herself to take care of them and make decisions about their care. Fumi has been taking a week's leave once every few months to stay with them. Fumi feels ambivalent about caring for her parents in the light of her desire to take good care of her daughter: ‘I feel bad because I am too busy caring for my parents and can't really give her proper attention’. Her ambivalent feeling is strong as she thinks she has done enough for her parents. Having supported her mother with her mental and physical illness since she was a teenager, Fumi would now rather focus on raising her child.

Fumi says, ‘whenever my mobile rings, I get nervous, suspecting that something has happened to my parents’. This is because one phone call may lead her to put her daily life on hold, go to her parent's house to provide care, and arrange and make decisions about their care as she is considered the main family carer. The policy context increases Fumi's ambivalence formed by temporal family relationships and gendered social expectation of childcare. The LTCI scheme helps her manage care for her parents remotely, but it regards her as the main carer. The responsibility of her parents' care interrupts her child's routine: ‘she wants to do her things like seeing her friends and going to her nursery’. Fumi's husband ‘supports’ her in taking care of her parents, listening to her complaints, and visiting while she and her daughter are at her parents' house. Fumi wonders how long this kind of life will last and says that the care and support she has to give to her parents prevents her from raising her daughter ‘properly’, which she worries may have an adverse influence on her development.

## 6 Entangled Ambivalence and Sandwich Care

Women's experience of caring simultaneously for children and parents has been under‐researched in the sociology of family and care. The existing research on sandwich care tends to focus on its problems and has detailed the difficulties and stress and their negative impact on carers' well‐being (Rubin and White‐Means 2009; Halinski et al. 2018). We have developed a theoretical approach to deepen our understanding of the sandwich carers' experience. Our approach to ambivalence highlights three of its properties: family relationships extending from the past into the future (temporal), the present contexts of social expectations (social structured) and social arrangement of care (policy‐contextual).

Sandwich carers' ambivalence arises from their everyday experience and negotiations required to cope with the multiple demands of care, and can be characterised by the entanglements of the three properties of ambivalence. Social expectations of care are inextricably bound up with sandwich carers' temporal family relationships. Ambivalence arises from trying to simultaneously fulfil the expected role as a daughter to meet the care needs of parents and as a mother to meet the care needs of children. Our analysis revealed that sandwich carers' negotiations with these competing social expectations and demands intersect with the past and future family care relationships, as illustrated through the selected carers' experiences. Role expectations and the willingness to respond to them as mothers and daughters are influenced by the relationships with parents that have been formed over time from the past, the relationships with children that have implications for the future, and by decisions and experiences associated with each relationship (such as quitting work to raise children or being asked by a deceased father to care for a mother).

Social expectations of motherhood strongly influence the ambivalence of sandwich carers. Sandwich carers' ambivalence can be understood with the motherhood ideology that providing care for their parents conflicts with them performing the expected mother's role of developing their children's characters. Emi left her job with the idea that mothers should look after their children when they are young. She is not an exception: 30.5% of Japanese women withdrew from the labour market on becoming a mother for the first time (Ministry of Health and Labour and Welfare 2022). A key reason for this is the demand for mothers to do ‘good parenting’, with particular emphasis on mother's responsibility for her child's education and discipline (Honda 2008; Shinada 2016). This demand is shared by those mothers in employment, as in Tomoko's case. The ‘educating family’, which undertakes to educate their children at home to develop their character and help them acquire academic qualifications for their financially and socially ‘secure’ future, has been the norm since the 1970s after the rapid economic development (Hirota 1999). Ohinata (2015) defines motherhood ideology, emphasising the uniqueness and centrality of the maternal role in a child's development, as a form of oppression which forces women to prioritise being a mother above all else. The lived experience of motherhood ideology was evident across our data. Motherhood ideology in contemporary neoliberal societies has been extensively studied, especially in terms of how women combine motherhood and paid work (for Japan, see Nukaga and Fujita 2022; Motohashi 2021). Our study adds unique findings on how motherhood ideology and family responsibility for adult social care intersect with women's negotiations with everyday sandwich care. The absence of a husband's engagement with care is often not considered a problem (except in Tomoko's case), indicating the sustained gendered pattern of sandwich care. This intensifies the ambivalence of women sandwich carers who feel that their engagement with older adult care prevents them from doing mothering ‘properly’.

Past family caring relationships also intersect with sandwich carers' ambivalence. Except for Fumi, who feels she has provided ‘enough’ care to her parents, the interviewees view caring for their parents as respecting their parents' wishes (Tomoko) or paying back what they owe from their past family relationships (Mai and Emi). Song's (2014) argument concerning South Korean sandwich carers may also apply to Fumi's case ‐ that ambivalent feelings are strongest when the carers provide care for parents out of a sense of obligation, even though they wish to prioritise childcare. For others, however, the experience of good family caring relationships in the past contributes to their ambivalence, as it strengthens their perceptions of the social expectation of the family's role in older parents' care. This somewhat paradoxical relationship—‘good’ family caring relationships in the past contributing to sandwich carers' ambivalence—was another key finding of our project.

Finally, social arrangements of care—care policy and the quality, accessibility, and availability of care services—also intersect with socially structured and temporal qualities of ambivalence in sandwich care. For all interviewees, publicly‐funded care services enable them to ‘manage’ sandwich care, even with long working hours (Tomoko). For Mai and Emi, both motherhood ideologies and family‐centred care responsibility (socially structured) and good caring relationships in the past (temporal) make them reluctant to use the care services, and both using and not using available services contribute to their ambivalence. Tomoko and Fumi use most of the care services available as they live away from their parents and cannot provide daily care. The policy frameworks increase their ambivalence: the policy framework expects the family to arrange care (Fumi) and to finance care (Tomoko). For Tomoko, the financial concerns regarding her father's future care cost drive her to take on part‐time work, which reduces her current available time and money for children, forming her ambivalence. Despite Fumi's wish not to be involved in caring for her parents, the LTCI scheme considers her their main carer and requires her to take responsibility. Social policy provides both material and ideological conditions and choices where people engage with care (Ungerson 1983). The sustained centrality of family and gendered expectations of care in Japanese social policy has been well discussed (Shire and Nemoto 2020; Liu and Yamashita 2020). Our analysis reveals that the social arrangement of care can mitigate or increase the social structured and temporal properties of ambivalence, with both also influence the access to and the experience of using services. Our findings therefor add unique findings that ideological conditions (based on carers' temporal and social structured conditions) also shape the use of available care services. These three qualities of ambivalence thus intersect in multiple ways. How they intersect requires further research, but our analysis indicates that social structured‐ especially motherhood ideology‐ and temporal qualities are at the core of ambivalence. This provides further evidence that the expansion of public care services should carefully consider local contexts of family and gendered responsibility of care to facilitate the just distribution of caring responsibility (Kurowska 2018).

We suggest that our theoretical approach to ambivalence can unpack the interweaving influences that shape the experience of sandwich carers and how they navigate everyday sandwich care. While our analysis focused on the context of Japan, the theoretical framework to ambivalence can be extended to other societies. Where ambivalence arises and what configuration of family relationships, social expectations and social arrangements of care create ambivalence, for example, is likely to differ in societies with little publicly funded childcare or adult social care services (the U.S.), where the social norm of filial piety exists and is practiced (China), or where both parents typically share childcare more equally (Sweden). Even in such different social, cultural, and policy contexts, the sociological ambivalence will serve to illustrate experiences for the increasing segment of the population engaging with sandwich care.

## Ethics Statement

The research project was conducted following the Codes of Ethics of the Japanese Sociological Society (https://jss‐sociology.org/about/researchpolicy/).

## Consent

The authors have nothing to report.

## Conflicts of Interest

The authors declare no conflicts of interest.

## Permission to Reproduce Material From Other Sources

The authors have nothing to report.

## Data Availability

The data that support the findings of this study are available on request from the corresponding author.

## Endnotes

^1^
Care managers are professionals who prepare care service plans for older people judged to be in need of care under the LTCI system. Based on the needs assessment and the older person's and their family's wish, a care manager develops, monitors and adjusts a care plan.

^2^
User's payment for the LTCI services is from 10 to 30 per cent of the total service cost depending on the household income.
